# Increased risk of cardiac death with cumulative exposure to fluid overload and dialysate sodium ≤138 mmol/l in hemodialysis patients

**DOI:** 10.1093/ckj/sfaf259

**Published:** 2025-08-19

**Authors:** Martin Christa, Brendan Smyth, Kaitlin J Mayne, Stefano Stuard, Bernard Canaud, Bernd Genser, Jule Pinter

**Affiliations:** Department for interal Medicine, University Hospital Würzburg, Würzburg, Germany; Heart-Lung Center, Department for Internal Medicine, Hospital Northwest, Frankfurt am Main, Germany; NHMRC Clinical Trials Centre, University of Sydney, Camperdown, NSW, Australia; Department of Renal Medicine, St George Hospital, Kogarah, NSW, Australia; University of Glasgow, Glasgow, UK; Global Medical Office, FMC Germany, Bad Homburg, Germany; NHMRC Clinical Trials Centre, University of Sydney, Camperdown, NSW, Australia; Department for interal Medicine, University Hospital Würzburg, Würzburg, Germany; High5Data GmbH, Heidelberg, Germany; Department for interal Medicine, University Hospital Würzburg, Würzburg, Germany

**Keywords:** cardiovascular, chronic hemodialysis, dialysis dose, fluid overload, prognosis

## Abstract

**Background:**

Fluid overload (FO) is an established risk factor for mortality in hemodialysis patients, who face higher cardiovascular mortality risk than the general population. Despite the known impact of FO on cardiovascular outcomes, the effects of cumulative FO exposure, dialysate sodium ≤138 mmol/l, and specific cardiac deaths remain unclear. This study aimed to assess these relationships in a large cohort of hemodialysis patients.

**Methods:**

This historical cohort study included 68 196 hemodialysis patients from the NephroCare network with data from 2010 to 2019. Eligible patients had valid bioimpedance, plasma, and dialysate sodium measurements within 90 days of starting hemodialysis. FO was defined as >7% relative FO, as assessed by bioimpedance spectroscopy. Cause-specific Cox proportional hazards models were used to evaluate the impact of these exposures on different cardiac endpoints, including death from heart failure, sudden cardiac death, and fatal myocardial infarction.

**Results:**

In 68 196 patients, a total of 2 123 957 patient-months were analyzed. Compared to patients with no prior exposure (0 month cumulative past exposure time), increasing cumulative exposure to FO (measured in months) significantly increased the risk of death from heart failure [hazard ratio (HR) peaking at 4.4, 95%CI: 3.4–5.6], sudden cardiac death (HR peaking at 4.0, 95%CI: 3.1–5.2), and fatal myocardial infarction (HR peaking at 3.3, 95%CI: 2.5–4.4). Cumulative exposure (in month) to dialysate sodium ≤138 mmol/l was associated with an elevated risk of death from heart failure (HR peaking at 6.0, 95%CI: 1.9–18.3) and a moderate increase in sudden cardiac death (HR peaking at 2.7, 95%CI: 1.3–5.6). The risk of all-cause mortality was consistently higher in patients exposed to FO (HR peaking at 3.4, 95%CI: 3.1–3.8) and those exposed to dialysate sodium ≤138 mmol/l (HR peaking at 2.0, 95%CI: 1.3–3.1).

**Conclusion:**

Cumulative FO and dialysate sodium ≤138 mmol/l significantly increase cardiac death risk in hemodialysis patients, particularly from heart failure. Stringent fluid management and careful consideration of dialysate sodium prescription is crucial to reduce cardiovascular mortality.

KEY LEARNING POINTS **What was known**:

- Fluid overload (FO) is a major risk factor for mortality in hemodialysis patients, who have a significantly higher cardiovascular death risk than the general population.
- While FO's impact on cardiovascular outcomes is well recognized, its cumulative effects over time and interaction with dialysate sodium levels remain unclear.
- The relationship between prolonged FO exposure, dialysate sodium ≤138 mmol/l, and specific cardiac causes of death has not been well studied.
- Prior research focused on general cardiovascular mortality, but understanding dose–response patterns for specific cardiac causes of death such as heart failure, sudden cardiac death, or myocardial infarction remains a critical gap.

**This study adds**:

- Prolonged fluid overload raises the risk of heart failure, sudden cardiac death, and fatal myocardial infarction.
- The linear exposure-risk pattern suggests that each single episode of fluid overload contributes to overall risk.
- Low dialysate sodium (≤138 mmol/l) was linked to a higher risk of heart failure-related death and suggests chronic sodium depletion may worsen myocardial stress and volume dysregulation.

**Potential impact**:

- A stringent cardioprotective fluid management is crucial to reduce cardiac mortality in hemodialysis patients, but regional variations in sodium prescriptions raise the need for additional randomized trials to optimize patient care.

## INTRODUCTION

Fluid overload (FO) is a well-established independent risk factor for mortality in patients undergoing hemodialysis [1, 2], who face a 10–20 times higher risk of (cardiovascular) mortality compared to the general population [3, 4].

This elevated risk is driven not only by atherosclerosis, but also by chronic hemodynamic stress, arrhythmogenic remodeling, and myocardial damage related to persistent fluid imbalance.

Recent data indicate that even mild FO (such as 1.1 l above dry weight) confers a stepwise increase in mortality, with cumulative exposure duration playing a critical role [2]. Similarly low dialysate sodium (≤138 mmol/l)—often the default setting in many centers—is associated with increased overall mortality, independent of plasma sodium and FO [5]. Thus, sodium concentration may modulate not only volume but also cardiac load. While lowering dialysate sodium may enhance sodium removal and reduce interdialytic weight gain, it can impair plasma refilling, increase the risk of intradialytic hypotension, and potentially worsen end-organ perfusion [6].

Although our prior analyses for overall mortality suggested that the mortality risk associated with FO is not modified by dialysate sodium, the impact of these exposures on specific cardiac causes of death remains unclear.

We hypothesized that prolonged exposure to FO and low dialysate sodium may contribute additively to cardiac mortality in hemodialysis patients. This study builds on our previous work by leveraging monthly, time-varying exposure data in a large international cohort to refine cardiovascular risk stratification by examining distinct cardiac death subtypes in relation to fluid and sodium balance over time.

## MATERIALS AND METHODS

### Study design

Data were obtained from NephroCare, a global dialysis network managed by Fresenius Medical Care, which operates in 25 countries throughout Europe, Africa, the Middle East, and Latin America. The electronic medical records of the patients treated at these centers are stored in the European Clinical Database 5 (EuCliD 5), a real-time electronic health record system tailored for routine dialysis care. To ensure high-quality data collection, updates are made daily, with monthly clinical governance checks. Ethical approval for this retrospective analysis was granted by the University Hospital in Würzburg, Germany (reference number 255/22).

### Study population

Hemodialysis patients were included from 1 January 2010 to 4 December 2019, if they had one valid plasma sodium and dialysate sodium and bioimpedance measurement within 90 days of starting hemodialysis. Most dialysis units across NephroCare use standardized dialysate sodium concentration with minimal variation across regions [2]. However to avoid bias by indication [7] we excluded patients prescribed profiling (whether of dialysate sodium and/or ultrafiltration; please see Supplementary Fig. S1). Follow-up began after the first available bioimpedance measurement and continued until death, transplantation, modality change, site change, or administrative censoring (4 December 2019).

### Exposures and covariates of interest

Fluid status was assessed pre-dialysis by bioimpedance spectroscopy using the body composition monitor in the supine position. This provided a more objective assessment than clinical evaluation [8]. Fluid status was based on average weekly fluid status (in liters) related to extracellular water (ECW in liters) and expressed as a percentage relative FO, to better reflect individual variance and to allow comparisons between individuals. NephroCare advises mid-week body composition measurements (BCM), but EuCliD records varied. We used “average weekly pre-dialysis fluid status” to standardize comparisons. This measure compensates for fluctuations between weekdays by subtracting 0.4 l on the first dialysis day (after the 2-day dialysis-free interval) and adding 0.2 l on the other days. These adjustments are based on internal EuCliD analyses (unpublished) that have determined compensation values close to 0.4 l as optimal for taking the “weekend surplus” into account. This approach smoothens a time series of fluid measurements by calculating moving averages. The equation for relative overhydration that we use was validated and corrected for body mass index to reduce systematic errors associated with extremes in body composition, thus enhancing the reliability of fluid volume measurements [9]. BCM are mandatory in NephroCare Clinics and not limited to a specific subset of patient (e.g. suspicious of FO).

FO was defined as any relative FO >7%. Several studies [10–13] have shown that moderate FO (>13%) is associated with an increased cardiovascular risk. A stricter threshold of 7% (∼1.1 l) was chosen because, according to recent studies [2], it is more effective at accurately identifying and quantifying lifetime exposure risk in dialysis patients.

If you translate the percentage into liters, it would be an approximation of +1.1 l excess for a typical adult with an average body weight of 70 kg.

The second exposure variable was dialysate sodium concentration, categorized as ≤138 and >138 mmol/l [5]. This threshold is based on our previous analyses using data from the EuCliD network, which demonstrated its clinical relevance and association with increased mortality risk. Dialysate sodium ≤138 mmol/l was the most frequently prescribed concentration (63.2%) and was independently associated with higher all-cause mortality [adjusted hazard ratio (HR) 1.57; 95% CI, 1.25–1.98], regardless of plasma sodium levels and other confounders [5]. Given these findings, we considered ≤138 mmol/l as the exposed group, reflecting real-world clinical practice and its potential long-term impact on patient outcomes.

BCM fluid status was mostly available monthly (mean = 0.9 measurements per month, standard deviations (SD) = 1.5), dialysate sodium was recorded for every dialysis session. Covariates were selected based on conceptual frameworks defined in previous work [5, 14] that aimed to classify all factors influencing mortality as risk factors, confounders, mediators, or effect modifiers. An overview of the conceptual framework used for epidemiological modeling is presented in the Supplement (see Fig. S2a and b).

### Endpoints

We considered the following exploratory endpoints presumed to reflect different—but admittedly sometimes overlapping—cardiac risk pathways that were defined based on the ICD10 codes listed in the EuCliD database as causes of death (specific ICD10 codes for each endpoint, see Supplement Table S1):

(i) death by heart failure (CHF),
(ii) sudden cardiac death and
(iii) death by fatal myocardial infarction (MI).

Additionally,

(iv) all-cause death was analyzed to compare the cause-specific effect estimates to the effect estimates on all-cause mortality.

For deeper understanding, we also performed our analyses for

(v) death by arrhythmia and
(vi) a composite of all cardiac fatal events (combining all listed ICD codes).

### Statistical analysis

Distributions of study variables were explored calculating descriptive statistics including means and SDs for continuous variables and frequencies for categorical variables. For skewed non-normally distributed variables median and interquartile ranges were calculated.

Many variables changed their values during the follow-up; therefore, we calculated in addition descriptive statistics of study characteristics based on patient-months (Table S5).

Time-to-event analysis using cause-specific Cox proportional hazard models including time-varying variables were used to estimate the effects of cumulative exposure time of the longitudinal exposure processes: namely, (i) FO, (ii) fluid depletion, and (iii) dialysate sodium ≤138 mmol/l. All models were fitted stratified by country and adjusted for clustering by medical center using a robust sandwich estimator.

The cause-specific hazard model considers only the failures from the cause of death of interest as events whereas failures from other causes were considered as censored observations. Exposure processes were coded by time-varying binary variables classifying each patient-month into the three risk conditions shown before (FO, fluid depletion, or dialysate sodium ≤138 mmol/l). Then we calculated for each patient-month three time-varying history variables counting the cumulative number of previous months spent in the respective exposure state.

First, we fitted univariate models for each of the exposure processes separately. Because a patient can have both past periods of FO and fluid depletion, we adjusted even in the univariate model the complementary process, i.e. fluid depletion was adjusted in the model of FO and vice versa. 

Second, multivariate core models including all confounding variables but excluding mediators according to our conceptual model (Supplement Fig. S2a and b). Third, we fitted a full multivariate model including all exposure processes with the pooled set of confounding variables. Finally, we included an interaction term in the full multivariate model to test whether there is effect modification between FO and low dialysis salt. In addition, we conducted sensitivity analyses to explore a selection bias due to premature death of high-risk subjects with high exposure intensity recalculating the models in the subpopulation of patients who survived 48 months (see for example the effect of FO on death from heart failure Supplement Table S2). Time-varying variables were evaluated using a monthly grid, the time series of the time-varying covariates used for the Cox regression models were smoothed by 3-month moving averages. When multiple measurements were available within a month the average of this measurements was calculated; if no measurement was recorded within a month, the last valid observation from the previous months was carried forward. For covariates missing in the first month cross-sectional mean imputation was used. Due to the size and complexity of the dataset, multiple imputation was not feasible.

Regarding potential confounding by dialysis vintage, we note that our study cohort includes only incident hemodialysis patients whose follow-up began within 90 days of dialysis initiation. Since dialysis vintage is strongly correlated with follow-up time in our study population (or is the same in many patients), and follow-up time is used as the time scale in all Cox models, the baseline hazard absorbs the effect of the time scale, thus any bias related to dialysis vintage is effectively controlled. All statistical analyses were conducted using Stata (StataCorp. 2023. Stata Statistical Software: Release 18. College Station, TX, USA: StataCorp LLC).

### Exposure-risk curves

We used a scaled decile bar chart to visualize the dose–response relationship of cumulative exposure, an approach previously applied in high-impact clinical and epidemiological studies (e.g. Pinter *et al*. [5]; Genser *et al.* [15]). Patient-months with nonzero exposure were grouped into deciles, while those with zero exposure served as the reference category; bars depict HRs with 95% confidence intervals, positioned along the *x*-axis according to the median exposure within each decile to facilitate visual interpretation of the exposure-risk pattern.

The reference group includes all patient-months with zero cumulative exposure, typically occurring early in follow-up. For example, if a patient first develops FO in month 7, the preceding 6 months contribute to the zero-exposure reference group.

## RESULTS

The minimum follow-up duration was 30 days, with a median survival of 71 months (95% CI, 70–73), totaling 2 123 957 patient-months of observation. By the end of the study, 21 644 patients (32%) had died, 6298 (9%) had received a kidney transplant, and 25 636 (38%) remained on hemodialysis. The remaining 14 618 patients (21%) were censored for various reasons, most commonly due to transfer to another facility (see Supplemental Table S6 for full details).

A total of 2 123 957 patient-months from 68 196 hemodialysis patients were analyzed. Descriptive statistics of the distribution of study patients see Table 1. The majority of patients were male (61%) with a mean age at begin of follow-up of 63.7 years (SD = 15.0), primarily of white ethnicity (52%), although ethnicity was unrecorded or unknown in over 45% of patients. Mean dialysis vintage at the time of the first body composition measurement was 28.0 months (SD = 22.8 months). Most patients had either a fistula (30.9%) or a catheter (64.8%) for vascular access, with only a minority having a graft (0.8%) and only few patients (3.6%) having no data on their vascular access (Table 1).

**Table 1:** Baseline characteristics of the study population (*N* = 68 196 hemodialysis patients).

Age (years)	63.7 (15.0)^a^
Sex (males)	41 504 (60.9%)
Dialysis vintage at first BCM (days)	28.0 (22.8)
Ethnicity (%)	
Asian	541 (0.8%)
Black	959 (1.4%)
White	35 713 (52.3%)
Unknown	30 983 (45.4%)
BMI (kg/m²)	26.9 (6.0)
Systolic blood pressure pre-dialysis (mmHg)	140.5 (19.5)
Creatinine (mg/dl)	6.6 (2.4)
Hemoglobin (g/l)	97.2 (16.1)
CRP (mg/l), median (IQR)	11.0 (7.6)
Leukocytes (×109 cells/l)	7.2 (1.8)
Albumin (g/l)	36.9 (5.2)
Ferritin (ng/ml)	418.3 (378.9)
Phosphate (mg/dl)	4.5 (1.4)
Single-pool Kt/V	1.3 (0.3)
Ultrafiltration (ml)	1 457 (1 300)
Vascular access (at baseline)	
Fistula	21 050 (30.9%)
Catheter	44 179 (64.8%)
Graft	538 (0.8%)
Unknown/other	2 429 (3.6%)
Comorbidities (history of)	
Diabetes	20 127 (29.5%)
Liver disease	4 147 (6.1%)
Cardiovascular disease	21 434 (31.4%)
Peripheral vascular disease	1 125 (1.7%)
Heart failure	7 662 (11.2%)
Malignancy	20 372 (29.9%)
Dementia	721 (1.1%)
Connective tissue disease	3 877 (5.7%)
Chronic lung disease	2 697 (3.9%)

^a^Data are mean (SD) or patient counts (%) of study variables. All study variables were evaluated at the begin of follow-up. Abbreviation: CRP = C reactive protein.

Up to one-third of patient-months during follow-up were associated with a history of diabetes (25.5%), malignancy (30.7%), or cardiovascular disease (35.4%). Heart failure was recorded in 11.9% of patient-months, while liver disease and chronic lung disease were less common, at 7.0% and 4.1%, respectively. Over the follow-up patients had a mean single-pool Kt/V ratio (dialysis efficacy) of 1.4 and a mean net ultrafiltration volume of 1952 ml. Patients spent 61% of months in FO and the mean cumulative number of FO exposure months per patient during the follow-up was 15.6 (SD = 17.1). Patients spent 64% of follow-up months on dialysate sodium ≤138 mm/l and the mean cumulative number of exposure months was 16.4 (SD = 20.4).

### Impact of abnormal fluid status on cardiac mortality

**Figure 1:**
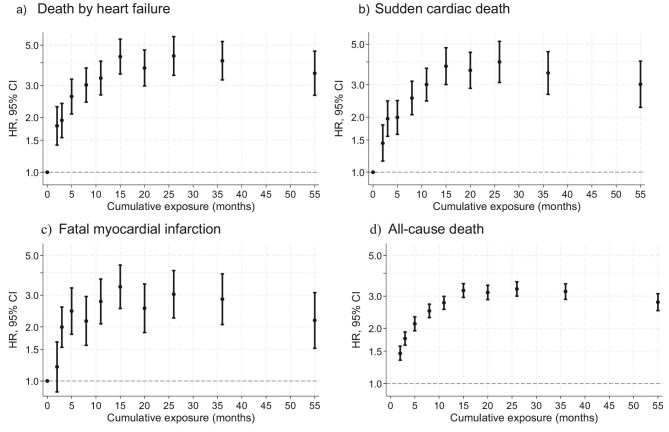
Impact of cumulative exposure time of FO on cause-specific hazards of fatal events: (**a**) death by heart failure (3248 events), (**b**) sudden cardiac death (2815 events), (**c**) fatal MI (1636 events), and (**d**) all-cause death (21 644 events). The medians of the quantile groups of cumulative exposure time are shown on the *x*-axes and the hazard ratios of cause-specific death with respect to the reference category ‘no-exposure (= 0)’ on the *y*-axes. Due to tied observations, it was not always possible to extract decile groups and the resulting number of groups with similar observation count are shown. Decile groups of patient-months with respect to cumulative exposure time (months) of any degree of relative FO are arranged on the *x*-axis according to the decile median exposure. A threshold of 7% defines any FO of >1.1 l above normal fluid status. The *y*-axis shows estimates of hazard ratios and 95% confidence intervals on a log scale as compared to the reference category (= 0 months of FO). Estimates are adjusted for the complementary exposure process ‘fluid depletion’ and age, sex, body mass index, ethnicity, concomitant diseases [diabetes mellitus, liver disease, heart failure, cardiovascular disease, cancer, dementia, connective tissue disease, chronic lung disease, serum creatinine, leukocytes, ferritin, hemoglobin, vascular access, and Kt/V (dialysis efficacy)].

Prolonged FO significantly increases the overall risk of death regardless of the cause (HR peak after 15 months: 3.4, 95%CI: 3.1–3.8, HR plateau 3.0). The exposure-risk pattern is most pronounced for death by heart failure (HR peak 4.4, 95%CI: 3.4–5.6 after 15 months, HR plateau ∼4.0), followed by sudden cardiac death with HR peaking at 4.0 (95%CI: 3.1–5.2) after 26 months and remaining consistently elevated. In comparison the exposure-risk pattern for fatal MI shows more variance with HR peaking at 3.3 (95%CI: 2.5–4.4) after 15 months and stabilizing ∼3.0 (Fig. 1). No significant risk pattern for FO we observed for death by arrythmia, whereas the pattern for all fatal cardiac events corroborates the effect of FO on cardiac risk (Fig. S3).

Cumulative exposure of fluid depletion also showed an increased risk for death from heart failure and sudden cardiac death but the association was less striking than for FO (Fig. S2).

### Impact of dialysate sodium ≤138 mmol/l on cardiac mortality

Dialysate sodium ≤138 mmol/l consistently increases the risk of cardiac death with HR peaking at 2.5 (95%CI: 2.5–4.4). The hazard ratio for death from heart failure shows some variation but increases over 40 months up to a peak HR of 6.0 (95%CI: 1.9–18.3), indicating that dialysate sodium ≤138 mmol/l is highly associated with death from heart failure.

**Figure 2:**
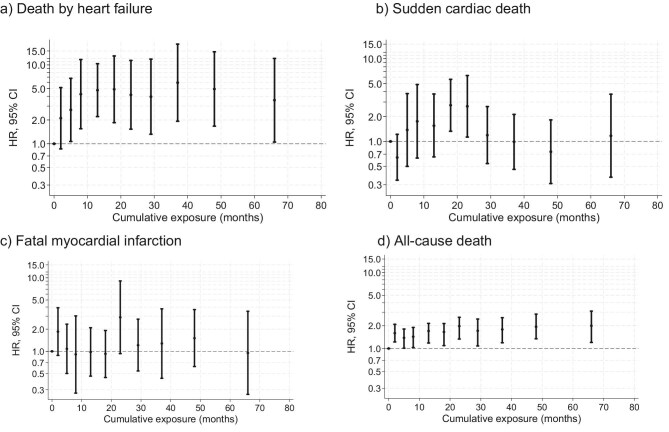
Impact of cumulative exposure time of dialysate sodium ≤138 mmol/l on cause-specific hazards of fatal events: (**a**) death by heart failure (3248 events), (**b**) sudden cardiac death (2815 events), (**c**) fatal MI, and (**d**) all-cause death (21 644 events). The medians of the quantile groups of cumulative exposure time are shown on the *x*-axes and the hazard ratios of cause-specific death with respect to the reference category ‘no-exposure (= 0)’ on the *y*-axes. Due to tied observations, it was not always possible to extract decile groups and the resulting number of groups with similar observation count are shown. Decile groups of patient-months with respect to cumulative exposure time (months) of dialysate sodium ≤138 are arranged on the *x*-axis according to the medians exposure of the decile. cumulative exposure time (months) of dialysate sodium ≤138 mmol/l. The *y*-axis shows hazard ratios and 95% confidence intervals on a log scale as compared to the reference category (>138 mmol/l). Estimates are adjusted for age, sex, body mass index, ethnicity, concomitant diseases [diabetes mellitus, liver disease, heart failure, cardiovascular disease, cancer, dementia, connective tissue disease, chronic lung disease, serum creatinine, leukocytes, ferritin, hemoglobin, vascular access, and Kt/V (dialysis efficacy)].

Second, low dialysate sodium may be associated with an increased risk of sudden cardiac death (peak HR 2.7, 95% CI 1.3–5.6). There was no evidence of an association with fatal MI (Fig. 2). The hazard ratios for death by arrythmia did not show any significant association pattern, whereas the risk pattern for all fatal cardiac indicates an increased risk but no dose–response relationship (Fig. S5b).

### Full model

As the exposure-risk curves for both FO and low dialysate sodium were more closely mirrored in death by heart failure and sudden cardiac death, we explored the joint exposure on those two cardiac causes in multivariate adjustment models.

A full multivariable model estimating the effect of FO on death by chronic heart failure using different adjusting variables including dialysate sodium (Table S2c) and without dialysate sodium (Table S2b) nearly show identical hazard ratios and thus provide no evidence for any confounding effect of dialysate sodium.

The results of sensitivity analysis for the endpoint sudden death shown in Table S3 also did not show any evidence for confounding.

For both endpoints there was also no evidence for any effect modification, the interaction terms were not significant (death by chronic heart failure: *P *= .180, sudden cardiac death: *P *= .990). Thus, we conclude that the effect of FO on cardiac risk acts independently from the effect of dialysate sodium (≤138 mmol/l).

### Sensitivity analysis

In sensitivity analyses restricted to patients who survived at least 48 months (Fig. S6b), the association between cumulative FO exposure and death from chronic heart failure was markedly stronger compared to the overall cohort (Fig. S6a). While the main analysis showed a plateau in hazard ratios beyond 20 months, the restricted cohort demonstrated a nearly linear increase in risk with longer exposure. Hazard ratios exceeded eight for the highest exposure categories, indicating a substantial and persistent impact of cumulative FO on mortality risk.

## DISCUSSION

This large historical cohort study of >68 000 hemodialysis patients demonstrates a significant association between cumulative exposure time of FO and an increased risk of cardiac death. The risk associated with FO is most pronounced for heart failure-related mortality (HR peak at 4.2 after ∼15 months of exposure). Additionally, there is a notable risk associated with sudden cardiac death and, to a lesser extent, fatal MI. Prolonged exposure to dialysate sodium ≤138 mmol/l was also linked to an elevated risk of heart failure-related death (HR peak at 6.0 over a period of 40 months).

Our findings indicate that FO and dialysate sodium act independently on the cardiac risk. The risk is largely driven by FO and to a lesser extent, by dialysate sodium.

These findings corroborate the critical impact of fluid management and dialysate sodium levels on cardiac outcomes in this patient population.

Our analysis shows a strong association between FO and different causes of cardiac death. In the hemodialysis population complex biological mechanisms intertwine. FO increases intravascular volume, which exerts excessive pressure on the heart, contributing to left ventricular hypertrophy and increased myocardial wall stress, eventually leading to heart failure [16]. This relationship is further exacerbated by the repetitive nature of hemodialysis, where patients experience cycles of volume overload and depletion [17].

During dialysis different factors such as enhanced fluid and diffusive salt removal can cause intradialytic hypotension straining the cardiovascular system and creating a cycle of ischemia and reperfusion injury [3]. This reduction in blood flow exposes the myocardium to ischemia and reperfusion damage contributing to myocardial stunning: a temporary loss of contractile function in the heart due to repeated ischemic episodes [18–20]. Although the damage from a single dialysis session may only be minimal, the cumulative effect of repeated exposure during hemodialysis leads to fibrosis and worsening cardiomyopathy [21].

The cardiac risk increase that we observe in our study emphasizes that clinicians should prioritize strategies that minimize FO and closely monitor patients for signs of volume excess.

These findings highlight the need for further research to disentangle the complex interactions between fluid management, different dialysate sodium levels, and cardiac outcomes in hemodialysis patients. Future studies should concentrate on identifying FO early [2], identifying patients at risk, and evaluate the most effective interventions to reduce chronic FO and the chain of negative consequences. Different fluid management strategies should be evaluated, such as adapted treatment time, and the optimal ultrafiltration rate in important patient subgroups to reduce cardiac mortality and major cardiovascular events, such as congested heart failure. Management should be tailored to important effect modifiers such as gender and or comorbidities. These interventional approaches should either be properly evaluated in pragmatic randomized controlled trials or if deemed unfeasible in target trial emulations using large contemporary data sets.

The present study has several limitations that must be considered. Despite extensive adjustments, confounding by country-, and or clinic specific factors may remain as hemodialysis practice is driven by facility-level management, which is difficult to elucidate in observational analyses. Data were collected from a source that combines electronic health and administrative records without manual chart validation. Therefore, diagnostic coding may have varied across sites and should be interpreted accordingly. Other important variables were unavailable, such as ethnicity and cardiac risk markers such as Troponin or NT-proBNP, which would have helped with cardiac risk stratification. Therefore, causality cannot be established.

Bioimpedance spectroscopy used to assess fluid status has inherent limitations, as it can be affected by factors such as electrode placement, obesity, or muscle wasting [22], and does not account for nutritional status, inflammation, or comorbidities.

Survivor bias is a potential concern in our analysis, as early dropout of high-risk individuals may have led to an underestimation of the true association between exposure and adverse outcomes. This form of selection bias is well recognized in longitudinal studies, where patients with the highest vulnerability are more likely to experience early events or discontinue follow-up. The apparent attenuation or plateau of risk with longer exposure likely reflects this bias rather than a genuine reduction in harm. Thus, the observed risk estimates, particularly beyond the initial exposure period, should be interpreted as conservative.

Another limitation of our study is the narrow variability in dialysate sodium prescriptions within individual clinics and countries, restricting the ability to assess the physiological impact of sodium gradients on outcomes. While interregional variation exists—with some regions favoring lower sodium to manage volume overload, while others prioritize hemodynamic stability—the prescription of default concentrations tends to be the norm. Additionally, as we excluded profiling patients to reduce indication bias, generalization might be limited.

Given the observational nature of our analysis, causal inferences regarding sodium prescription remain limited. Ongoing randomized trials, such as RESOLVE (NCT02823821), are essential to determine whether dialysate sodium modification can improve cardiovascular outcomes.

A further limitation of our analysis is that the cause-specific hazard modeling approach assumes absence of bias due to a common and/or competing risk situation. A more complex competing risk models might be applied as additional sensitivity analyses by aiming to account for the bias due to an effect of competing underlying risk pathway(s). However unclear working hypothesis about the underlying common/competing complexity and small number of events for some endpoints make this analysis not feasible. Finally, although NephroCare operates under strict guidelines to ensure high data quality, there remains a possible bias due to misclassification of cause of death as classification is done by the treating clinician with no independent research driven adjudication or cross validation and therefore prone to human error. For example, a death caused by arrhythmia could be coded as sudden cardiac death, or a fatal MI might be recorded as the fatal arrhythmia it induced.

In conclusion, our study showed that cumulative exposure time of FO and dialysate sodium ≤138 mmol/l substantially affected the cardiac risk of hemodialysis patients, mainly death from heart failure. For FO, we identified a linear increasing hazard pattern with increasing cumulative exposure, reinforcing a dose–response relationship. The greater the exposure to FO, the higher the associated cardiac risk, and each individual episode of FO appears to contribute incrementally to the overall risk. This exposure-risk pattern suggests that even short-term episodes of FO are not benign but cumulatively increase the likelihood of adverse cardiac outcomes.

Our findings highlight the need for early and consistent fluid management to prevent irreversible complications. Future research should prioritize evaluating cardioprotective fluid strategies to reduce long-term cardiovascular risk in hemodialysis patients.

## Supplementary Material

sfaf259_Supplemental_File

## Data Availability

Due to data protection issues the full study dataset is not available on a public repository because it is an extraction of a real-time electronic health record system, namely the European Clinical Database 5 (EuCliD 5). An anonymized version of the study dataset is available upon reasonable request.
