# Supplementary material for: Increased risk of cardiac death with cumulative exposure to fluid overload and dialysate sodium ≤138 mmol/l in hemodialysis patients
Source: Clin Kidney J. 2025 Aug 19;18(10):sfaf259. doi: 10.1093/ckj/sfaf259 (PMC12541371; doi:10.1093/ckj/sfaf259)
Supplement: sfaf259_Supplemental_File [file sfaf259_supplemental_file.docx]

**SUPPLEMENTARY MATERIAL**

Table S1: Definition of endpoints and risk pathways

| **Endpoint** | **ICD10 code** | **Presumed underlying risk pathways** |
| --- | --- | --- |
| Death by arrythmia | I44, I45,  I47, I49 | chronic inflammation, repeated ischemic episodes, underlying structural heart disease, genetic predisposition, external factors (drugs or toxins) |
| Sudden cardiac death | I46 | abnormal blood pressure, chronic inflammation, (undiagnosed) inherited arrhythmia syndromes or cardiomyopathies, acute ischemic events, electrolyte imbalance, drug interaction (e.g. QT-prolonging drugs) |
| Death by chronic heart failure | I11, I13, I42, I43, I50,  I51 | atherosclerosis, chronic inflammation, increased cardiac fibrosis, neurohormonal activation (e.g., RAAS and sympathetic nervous system), systemic hypoperfusion, fluid retention, |
| Fatal myocardial infarction | I21, I22, I23, I25 | Atherosclerosis, chronic inflammation, urotoxins, hyperlipoprotinaemia,  Hypotension, repeated vessle wall shear stress due to volume fluctuations |
| All fatal cardiac events | I11, I13, I21, I22, I23, I24, I25, I42, I43, I44, I45, I46, I47, I49, I50, I51 | All the above mentioned ways |

TABLE S2: Sensitivity analysis: Results of cause specific hazard models estimating the effect of fluid overload on death by chronic heart failure using different adjusting variables (A: Univariate model, B: multivariate model, C: full multivariate model)

A)

| Decile | HR | 95% CI |
| --- | --- | --- |
| 1 | 1.92 | 1.52 - 2.43 |
| 2 | 2.20 | 1.78 - 2.71 |
| 3 | 3.20 | 2.58 - 3.97 |
| 4 | 3.88 | 3.12 - 4.82 |
| 5 | 4.51 | 3.64 - 5.60 |
| 6 | 6.34 | 5.07 - 7.92 |
| 7 | 5.75 | 4.54 - 7.27 |
| 8 | 7.05 | 5.45 - 9.13 |
| 9 | 7.39 | 5.73 - 9.54 |
| 10 | 7.27 | 5.44 - 9.72 |

B)

| Decile | HR | 95% CI |
| --- | --- | --- |
| 1 | 1.80 | 1.41 - 2.29 |
| 2 | 1.93 | 1.55 - 2.39 |
| 3 | 2.61 | 2.09 - 3.25 |
| 4 | 3.02 | 2.43 - 3.74 |
| 5 | 3.29 | 2.66 - 4.08 |
| 6 | 4.32 | 3.47 - 5.39 |
| 7 | 3.74 | 2.98 - 4.70 |
| 8 | 4.36 | 3.41 - 5.57 |
| 9 | 4.10 | 3.22 - 5.23 |
| 10 | 3.50 | 2.65 - 4.63 |

C)

| Decile | HR | LB 95% CI |
| --- | --- | --- |
| 1 | 1.80 | 1.41 - 2.29 |
| 2 | 1.93 | 1.55 - 2.39 |
| 3 | 2.61 | 2.10 - 3.25 |
| 4 | 3.02 | 2.43 - 3.74 |
| 5 | 3.29 | 2.66 - 4.08 |
| 6 | 4.33 | 3.47 - 5.40 |
| 7 | 3.74 | 2.98 - 4.70 |
| 8 | 4.36 | 3.40 - 5.58 |
| 9 | 4.10 | 3.21 - 5.22 |
| 10 | 3.52 | 2.66 - 4.65 |

Legend Table S2: Univariate model is adjusted only for the complementary process (fluid depletion). Multivariate model is additionally adjusted for age, sex, body mass index, ethnicity, concomitant diseases (diabetes mellitus, liver disease, chronic heart failure, cardiovascular disease, cancer, dementia, connective tissue disease, chronic lung disease, serum creatinine, leukocytes, ferritin, hemoglobin, vascular access and Kt/V [dialysis efficacy]). Full multivariate model is additionally adjusted for cumulative exposure time of low dialysate sodium.

TABLE S3: Sensitivity analysis: Results of cause specific hazard models estimating the effect of fluid overload on death by sudden cardiac death using different adjusting variables (A: Univariate model, B: multivariate model, C: full multivariate model)

A)

| Decile | HR | 95% CI |
| --- | --- | --- |
| 1 | 1.50 | 1.21 - 1.85 |
| 2 | 2.07 | 1.68 - 2.55 |
| 3 | 2.24 | 1.82 - 2.77 |
| 4 | 2.92 | 2.36 - 3.61 |
| 5 | 3.60 | 2.92 - 4.45 |
| 6 | 4.66 | 3.67 - 5.91 |
| 7 | 4.70 | 3.72 - 5.94 |
| 8 | 5.47 | 4.15 - 7.20 |
| 9 | 5.18 | 3.87 - 6.94 |
| 10 | 5.25 | 3.73 - 7.39 |

B)

| Decile | HR | 95% CI |
| --- | --- | --- |
| 1 | 1.44 | 1.15 - 1.81 |
| 2 | 1.96 | 1.57 - 2.45 |
| 3 | 1.99 | 1.61 - 2.46 |
| 4 | 2.54 | 2.06 - 3.14 |
| 5 | 3.01 | 2.45 - 3.69 |
| 6 | 3.79 | 3.01 - 4.78 |
| 7 | 3.60 | 2.87 - 4.51 |
| 8 | 4.00 | 3.09 - 5.18 |
| 9 | 3.48 | 2.66 - 4.55 |
| 10 | 3.02 | 2.26 - 4.04 |

C)

| Decile | HR | LB 95% CI |
| --- | --- | --- |
| 1 | 1.44 | 1.15 - 1.81 |
| 2 | 1.96 | 1.57 - 2.45 |
| 3 | 2.00 | 1.61 - 2.47 |
| 4 | 2.54 | 2.06 - 3.14 |
| 5 | 3.01 | 2.46 - 3.70 |
| 6 | 3.79 | 3.01 - 4.78 |
| 7 | 3.59 | 2.86 - 4.49 |
| 8 | 4.00 | 3.09 - 5.18 |
| 9 | 3.49 | 2.66 - 4.56 |
| 10 | 3.03 | 2.26 - 4.05 |

Legend Table S3: Univariate model is adjusted only for the complementary process (fluid depletion). Multivariate model is additionally adjusted for age, sex, body mass index, ethnicity, concomitant diseases (diabetes mellitus, liver disease, chronic heart failure, cardiovascular disease, cancer, dementia, connective tissue disease, chronic lung disease, serum creatinine, leukocytes, ferritin, hemoglobin, vascular access and Kt/V [dialysis efficacy]). Full multivariate model is additionally adjusted for cumulative exposure time of low dialysate sodium.

Table S4: Distribution of patients among the countries participating in the European clinical database.

| Country | No. of sites | N (%) |
| --- | --- | --- |
| AR | 95 | 4,065 (6.47) |
| BA | 10 | 1,022 (1.42) |
| BR | 38 | 3,140 (4.51) |
| CL | 62 | 2,312 (3.47) |
| CO | 43 | 6,620 (10.23) |
| CZ | 27 | 1,744 (2.53) |
| EC | 16 | 1,745 (2.44) |
| EE | 15 | 206 (0.31) |
| FR | 36 | 2,079 (3.47) |
| HR | 06 | 0359 (0.51) |
| HU | 23 | 2,287 (3.24) |
| IT | 51 | 1,892 (2.79) |
| KZ | 04 | 6 (0.01) |
| PE | 03 | 14 (0.02) |
| PL | 73 | 8,717 (12.55) |
| PT | 40 | 3,144 (4.43) |
| RO | 35 | 6,342 (9.92) |
| RU | 76 | 5,865 (9.06) |
| SA | 32 | 0796 (1.16) |
| SK | 34 | 1,856 (2.70) |
| SL | 06 | 386 (0.55) |
| SP | 63 | 7,267 (10.36) |
| SR | 04 | 105 (0.15) |
| TR | 54 | 5518 (7.71) |
| UK | 29 | 709 (1.00) |
| Total | 875 | 68,196 (100.00) |

Table S4 legend: Abbreviations: AR = Argentina, BA = Bosnia, BR = Brazil, CL = Chile, CO = Colombia, CZ = Czech Republic, EC = Ecuador, EE = Estonia, FR = France, HR = Herzegovina, HU = Hungary, IT = Italy, KZ = Croatia, PE = Peru, PL = Poland, PT = Portugal, RO = Romania, RU = Russia, SA = South

Table S5: Descriptive statistics of 2,123,957 patient months in N=68,196 hemodialysis patients.

| Fluid overload months | | 1,298,752 (61.1%) |  |
| --- | --- | --- | --- |
| Fluid depletion months | | 80,922 (3.8%) |  |
| Months with dialysate sodium <= 138 mm/l | | 1,360,919 (64.1) |  |
|  |  | |  |
| BMI (kg/m²) | 27.4 (6.0) | |  |
| Systolic blood pressure pre-dialysis (mmHg) | 140.2 (20.3) | |  |
| Creatinine (mg/dl) | 7.3 (2.6) | |  |
| Hemoglobin (g/l) | 109.3 (15.7) | |  |
| CRP (mg/l), median (IQR) | 9.7 (8.1) | |  |
| Leukocytes (x 109 cells/l) | 7.0 (1.6) | |  |
| Albumin (g/l) | 38.8 (4.8) | |  |
| Ferritin (ng/ml) | 558.5 (432.2) | |  |
| Phosphate (mg/dl) | 4.6 (1.4) | |  |
| Single-pool Kt/V | 1.4 (0.3) | |  |
| Ultrafiltration (mL) | 1951.5 (1370.9) | |  |
| Comorbidities |  | |  |
| Diabetes | 541,213 (25.5%) | | |
| Liver Disease | 149,297 (7.0%) | | |
| Cardiovascular disease | 751,175 (35.4%) | | |
| Peripheral vascular disease | 42,250 (2.0%) | | |
| Heart failure | 252,891 (11.9%) | | |
| Malignancy | 652,075 (30.7%) | | |
| Dementia | 22,500 (1.1%) | | |
| Connective tissue disease | 128,315 (6.0%) | | |
| Chronic lung disease | 86,874 (4.1%) | | |

Legend Table S4: Data are mean (SD) or patient months (%) unless otherwise stated. All variables were evaluated as time-varying at a monthly grid (patient months). Comorbidities were considered as chronic diseases presuming persistent since the first diagnosis of the disease. Abbreviations: BCM = body composition measurement. CRP= C reactive protein. Single-pool Kt/V=dialysis efficacy.

Table S6: Reasons for censoring of N=20,916 patients

| Discharge reason | No. (%) |
| --- | --- |
| Modality change |  |
| 1. Home Hemodialysis | 192 (0.9%) |
| 1. Peritoneal Dialysis | 549 (2.6%) |
| Transplantation | 6298 (31.1%) |
| Recovery of renal function | 283 (1.4%) |
| Transfer to another site | 8175 (39.12%) |
| Lost to follow-up | 2014 (9.62%) |
| Treatment stopped | 1751 (8.4%) |
| Other | 1654 ((7.9%) |
| Total | 20,916 (100.0) |

Figure S1: Flowchart: Selection of study population.

Figure S2. Conceptual framework used for epidemiological modelling: A) Fluid overload, B) Dialysate sodium

A):
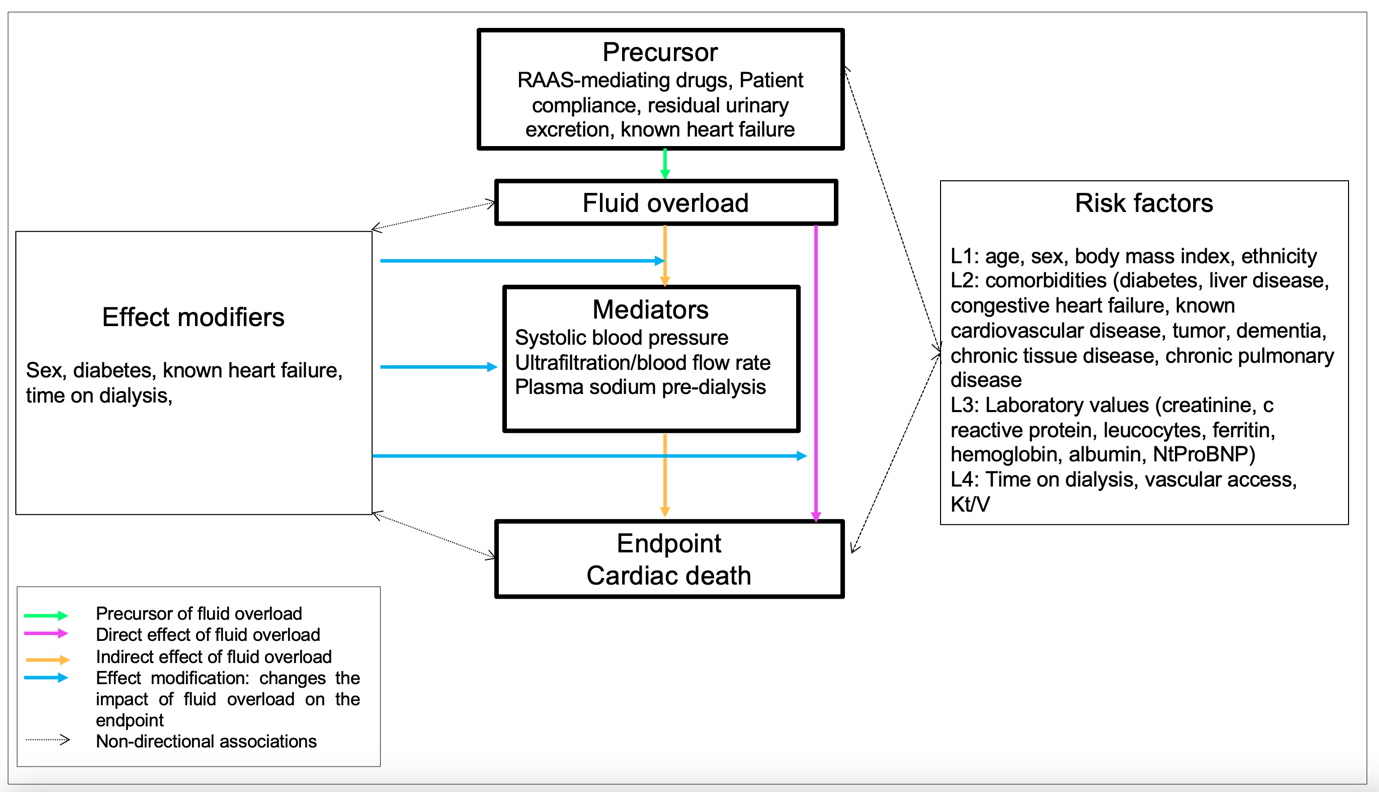


B):


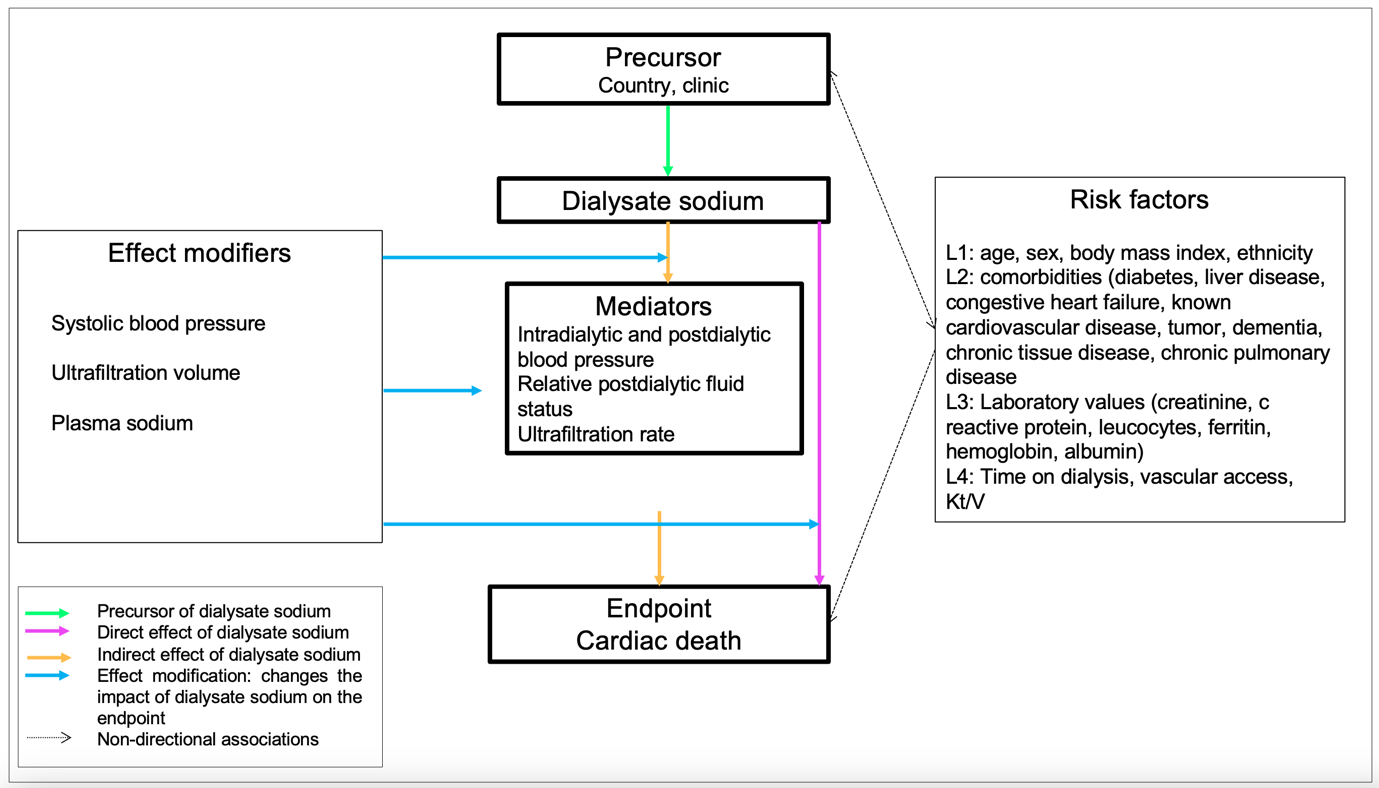


Figure S3: Impact of cumulative exposure time of fluid overload on cause specific hazard of fatal events (additional endpoints):

1. death by arrythmia (158 events), B) all fatal cardiac events (7,926 events)

A) death by arrythmia B) all fatal cardiac events

Legend Figure S1: X-axis shows medians of quantile groups of cumulative exposure (months) of fluid overload. Bars on y-axis show multivariate hazard ratios and 95% confidence intervals as compared to the reference category (= 0 months of fluid overload) adjusted for the complementary exposure process (=fluid depletion) and age, sex, body mass index, ethnicity, concomitant diseases (diabetes mellitus, liver disease, chronic heart failure, cardiovascular disease, cancer, dementia, connective tissue disease, chronic lung disease, serum creatinine, leukocytes, ferritin, hemoglobin, vascular access, and Kt/V [dialysis efficacy]).

Figure S4: Impact of cumulative exposure time of fluid depletion on cause-specific hazard of fatal events (A) death by chronic heart failure (3,248 events), B) sudden cardiac death (2,815 events), C) fatal MI (1,636 events events), D) all cause death (21,644 events), E) death by arrythmia (158 events), F) all fatal cardiac events (7,926 events)

A) death by chronic heart failure B) sudden cardiac death C) fatal MI

**

E) all cause death F) all fatal cardiac events

**

Legend Figure S2: X-axis shows medians of quantile groups of cumulative exposure (months) of fluid depletion. Bars on y-axis show multivariate hazard ratios and 95% confidence intervals as compared to the reference category (= 0 months of fluid depletion) adjusted for the complementary exposure process (=any degree of fluid overload) and age, sex, body mass index, ethnicity, concomitant diseases (diabetes mellitus, liver disease, chronic heart failure, cardiovascular disease, cancer, dementia, connective tissue disease, chronic lung disease, serum creatinine, leukocytes, ferritin, hemoglobin, vascular access, and Kt/V [dialysis efficacy]).

Figure S5. Impact of cumulative exposure time of low dialysate sodium on cause specific hazard of fatal events: A) death by arrythmia (158 events), B) all fatal cardiac events (7,926 events)

1. death by arrythmia B) all fatal cardiac events

**

Legend Figure S3: x-axis shows medians of decile groups of cumulative exposure time (months) of low dialysate sodium. Bars on y-axis show estimates of hazard ratios & 95% confidence intervals on a log scale as compared to the reference category (=0 months on low dialysate sodium). Estimates are adjusted for age, sex, body mass index, ethnicity, concomitant diseases (diabetes mellitus, liver disease, chronic heart failure, cardiovascular disease, cancer, dementia, connective tissue disease, chronic lung disease, serum creatinine, leukocytes, ferritin, hemoglobin, vascular access, and Kt/V [dialysis efficacy]).

Figure S6: Sensitivity analysis: Impact of cumulative exposure time of any fluid overload on death by chronic heart failure, A) all patients, B) subpopulation of patients that survived 48 months or longer.

1. death by CHF – all patients B) death by CHF - patients that survived 48 months or longer

**

Legend Figure 4S: x-axis shows medians of decile groups of cumulative exposure time (months) of any relative fluid overload. y-axis shows estimates of hazard ratios & 95% confidence intervals on a log scale as compared to the reference category (=0 months of severe fluid overload). Estimated hazard ratios are adjusted for the complementary process ‘fluid depletion’ and covariates age, sex, body mass index, ethnicity, concomitant diseases (diabetes mellitus, liver disease, chronic heart failure, cardiovascular disease, cancer, dementia, connective tissue disease, chronic lung disease, serum creatinine, leukocytes, ferritin, hemoglobin, vascular access, and Kt/V [dialysis efficacy]).
